# 3-[(2-Hy­droxy-1-naphth­yl)(pyrrolidin-1-yl)meth­yl]benzonitrile

**DOI:** 10.1107/S1600536810026152

**Published:** 2010-07-14

**Authors:** Meng Wei Xue

**Affiliations:** a Biochemical and Environmental Engineering College, Nanjing Xiaozhuang University, Nanjing 210017, People’s Republic of China

## Abstract

The title compound, C_22_H_20_N_2_O, was obtained from the condensation reaction of 3-formyl­benzonitrile, 2-naphthol and pyrrolidine. There are two mol­ecules in the asymmetric unit, having similar conformations. Intra­molecular O—H⋯N and C—H⋯O hydrogen bonds occur, with only van der Waals forces between mol­ecules. The dihedral angles between the naphthalene ring system and the phenyl ring in the two molecules are 75.28 (10) and 76.07 (11)°. The five-membered rings adopt half-chair conformations.

## Related literature

For the applications of Betti-type reactions, see: Lu *et al.* (2002 ▶); Xu *et al.* (2004 ▶); Wang *et al.* (2005 ▶).
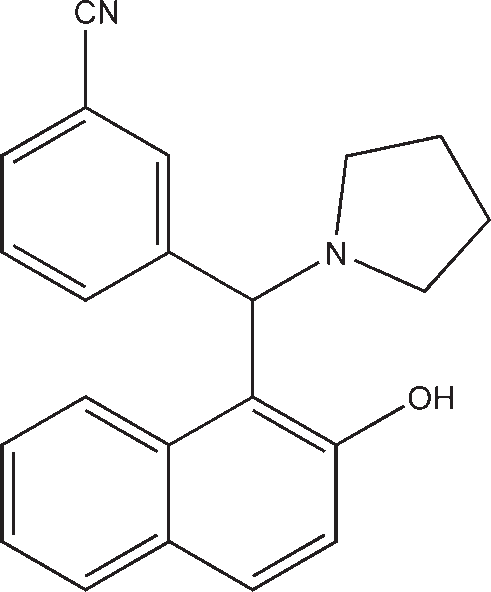


## Experimental

### 

#### Crystal data

C_22_H_20_N_2_O
*M* *_r_* = 328.40
Orthorhombic, 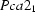

*a* = 18.735 (4) Å
*b* = 10.475 (2) Å
*c* = 18.122 (4) Å
*V* = 3556.4 (12) Å^3^
*Z* = 8
Mo *K*α radiation
μ = 0.08 mm^−1^
*T* = 293 K
0.20 × 0.20 × 0.20 mm

#### Data collection

Rigaku Mercury2 diffractometer
Absorption correction: multi-scan (*CrystalClear*; Rigaku, 2005 ▶) *T*_min_ = 0.825, *T*_max_ = 1.000
31413 measured reflections
3612 independent reflections
2166 reflections with *I* > 2σ(*I*)
*R*_int_ = 0.125

#### Refinement

*R*[*F* ^2^ > 2σ(*F* ^2^)] = 0.064
*wR*(*F* ^2^) = 0.149
*S* = 1.07
3612 reflections
452 parameters
1 restraint
H-atom parameters constrained
Δρ_max_ = 0.16 e Å^−3^
Δρ_min_ = −0.16 e Å^−3^

### 

Data collection: *CrystalClear* (Rigaku, 2005 ▶); cell refinement: *CrystalClear*; data reduction: *CrystalClear*; program(s) used to solve structure: *SHELXS97* (Sheldrick, 2008 ▶); program(s) used to refine structure: *SHELXL97* (Sheldrick, 2008 ▶); molecular graphics: *SHELXTL/PC* (Sheldrick, 2008 ▶); software used to prepare material for publication: *SHELXTL/PC*.

## Supplementary Material

Crystal structure: contains datablocks I, global. DOI: 10.1107/S1600536810026152/rz2464sup1.cif

Structure factors: contains datablocks I. DOI: 10.1107/S1600536810026152/rz2464Isup2.hkl

Additional supplementary materials:  crystallographic information; 3D view; checkCIF report

## Figures and Tables

**Table 1:** Hydrogen-bond geometry (Å, °)

*D*—H⋯*A*	*D*—H	H⋯*A*	*D*⋯*A*	*D*—H⋯*A*
O1—H1*A*⋯N1	0.82	1.90	2.576 (5)	139
O2—H2*A*⋯N3	0.82	1.93	2.593 (5)	138
C39—H39*A*⋯O2	0.93	2.58	3.292 (6)	133

**Fig. 1.**
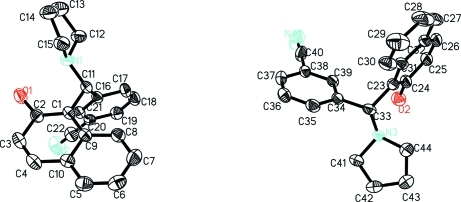
Perspective structure of the title compound, showing the atom-numbering scheme. Displacement ellipsoids are drawn at the 30% probability level.

## supplementary crystallographic information

### Comment

Over one hundred years ago, Betti developed a straightforward synthesis involving the condensation of 2-naphthol, ammonia and equivalents of benzaldehyde, followed by the addition of HCl and KOH to yield 1-(a-aminobenzyl)-2-naphthol. This product which possesses an asymmetric carbon center is known as a Betti base. Betti-type reaction is an important method to synthesize chiral ligands and by this method many unnatural homochiral amino-phenol compounds have been obtained (Lu *et al.* 2002; Xu *et al.* 2004; Wang *et al.* 2005). Here we report the synthesis and crystal structure of the title compound, 3-[(2-hydroxynaphthalen-1-yl)(pyrrolidin-1-yl)methyl]benzonitrile (Fig. 1).

Both molecules in the asymmetric unit have the same relative conformation at the chiral carbon atoms. The naphthalene (A; C1–C10, B; C23–C32) and benzene (C; C16–C21, D; C34–C39) rings are strictly planar and the dihedral angles between A/C and B/D are 75.28 (10) and 76.07 (11)°, respectively. The two molecules are stabilized by intramolecular O—H···N hydrogen bonding, whereas only one is involved in intramolecular C—H···O hydrogen bonds (Table 1). Intermolecular interactions are only van der Waals forces.

### Experimental

3-Formylbenzonitrile (1.97 g, 0.015 mol) and pyrrolidine (1.065 g, 0.015 mol) was added to 2-naphthol (2.16 g, 0.015 mol) without solvent under nitrogen. The temperature was raised gradually to 120°C in one hour and the mixture was stirred at this temperature for 12 h. The system was treated with 30 ml of ethanol 95% and cooled. The precipitate was filtered and washed with a small amount of ethanol 95%. The title compound was isolated using column chromatography (petroleum ether:ethyl acetate 4:1 *v*/*v*). Single crystals suitable for X-ray diffraction analysis were obtained by slow evaporation of an ethyl acetate solution at room temperature.

### Refinement

All H atoms were positioned geometrically and refined using a riding model, with C—H = 0.93–0.97 Å, O—H = 0.82 Å, and with *U*_iso_(H) = 1.2 *U*_eq_(C) or 1.5 *U*_eq_(O). In the absence of significant anomalous scattering effects, the 3377 Friedel pairs were merged. The relatively high *R*_int_ value and the low data/parameter ratio reflects the poor quality of the crystal.

### Crystal data

**Table:**

C_22_H_20_N_2_O	*F*(000) = 1392
*M**_r_* = 328.40	*D*_x_ = 1.227 Mg m^−^^3^
Orthorhombic, *P**c**a*2_1_	Mo *K*α radiation, λ = 0.71073 Å
Hall symbol: P 2c -2ac	Cell parameters from 3612 reflections
*a* = 18.735 (4) Å	θ = 2.6–26.0°
*b* = 10.475 (2) Å	µ = 0.08 mm^−^^1^
*c* = 18.122 (4) Å	*T* = 293 K
*V* = 3556.4 (12) Å^3^	Prism, colourless
*Z* = 8	0.20 × 0.20 × 0.20 mm

### Data collection

**Table:**

Rigaku Mercury2 diffractometer	3612 independent reflections
Radiation source: fine-focus sealed tube	2166 reflections with *I* > 2σ(*I*)
graphite	*R*_int_ = 0.125
Detector resolution: 13.6612 pixels mm^-1^	θ_max_ = 26.0°, θ_min_ = 3.1°
CCD_Profile_fitting scans	*h* = −23→23
Absorption correction: multi-scan (*CrystalClear*; Rigaku, 2005)	*k* = −12→12
*T*_min_ = 0.825, *T*_max_ = 1.000	*l* = −22→22
31413 measured reflections	

### Refinement

**Table:**

Refinement on *F*^2^	Primary atom site location: structure-invariant direct methods
Least-squares matrix: full	Secondary atom site location: difference Fourier map
*R*[*F*^2^ > 2σ(*F*^2^)] = 0.064	Hydrogen site location: inferred from neighbouring sites
*wR*(*F*^2^) = 0.149	H-atom parameters constrained
*S* = 1.07	*w* = 1/[σ^2^(*F*_o_^2^) + (0.065*P*)^2^ + 0.150*P*] where *P* = (*F*_o_^2^ + 2*F*_c_^2^)/3
3612 reflections	(Δ/σ)_max_ < 0.001
452 parameters	Δρ_max_ = 0.16 e Å^−^^3^
1 restraint	Δρ_min_ = −0.16 e Å^−^^3^

### Special details

**Table:**

Geometry. All e.s.d.'s (except the e.s.d. in the dihedral angle between two l.s. planes) are estimated using the full covariance matrix. The cell e.s.d.'s are taken into account individually in the estimation of e.s.d.'s in distances, angles and torsion angles; correlations between e.s.d.'s in cell parameters are only used when they are defined by crystal symmetry. An approximate (isotropic) treatment of cell e.s.d.'s is used for estimating e.s.d.'s involving l.s. planes.
Refinement. Refinement of *F*^2^ against ALL reflections. The weighted *R*-factor *wR* and goodness of fit *S* are based on *F*^2^, conventional *R*-factors *R* are based on *F*, with *F* set to zero for negative *F*^2^. The threshold expression of *F*^2^ > σ(*F*^2^) is used only for calculating *R*-factors(gt) *etc*. and is not relevant to the choice of reflections for refinement. *R*-factors based on *F*^2^ are statistically about twice as large as those based on *F*, and *R*- factors based on ALL data will be even larger.

### Fractional atomic coordinates and isotropic or equivalent isotropic displacement parameters (Å^2^)

**Table:**

	*x*	*y*	*z*	*U*_iso_*/*U*_eq_	
O1	0.99425 (19)	0.9948 (4)	0.6386 (2)	0.0722 (11)	
H1A	0.9848	0.9279	0.6173	0.108*	
O2	0.1596 (2)	0.5126 (4)	0.3506 (2)	0.0758 (11)	
H2A	0.1799	0.5805	0.3587	0.114*	
C20	0.8916 (3)	0.9893 (4)	0.4063 (3)	0.0511 (12)	
C34	0.2940 (2)	0.6121 (4)	0.4724 (3)	0.0481 (11)	
C1	0.8658 (3)	1.0060 (5)	0.6434 (3)	0.0506 (12)	
N3	0.1789 (2)	0.7119 (3)	0.4331 (2)	0.0556 (11)	
C17	0.8022 (3)	0.8267 (5)	0.4799 (3)	0.0613 (13)	
H17A	0.7717	0.7707	0.5044	0.074*	
C16	0.8526 (2)	0.8953 (4)	0.5204 (3)	0.0478 (11)	
N1	0.9174 (2)	0.7925 (4)	0.6241 (2)	0.0553 (11)	
C35	0.3411 (3)	0.6798 (5)	0.5162 (3)	0.0649 (14)	
H35A	0.3232	0.7354	0.5516	0.078*	
C23	0.1768 (2)	0.4942 (4)	0.4814 (3)	0.0540 (12)	
C21	0.8972 (2)	0.9772 (4)	0.4825 (3)	0.0511 (12)	
H21A	0.9312	1.0245	0.5081	0.061*	
C33	0.2139 (2)	0.6242 (4)	0.4850 (3)	0.0512 (12)	
H33A	0.2068	0.6583	0.5348	0.061*	
C2	0.9328 (3)	1.0546 (5)	0.6582 (3)	0.0560 (13)	
C9	0.8046 (3)	1.0760 (5)	0.6660 (2)	0.0495 (12)	
C39	0.3220 (2)	0.5309 (4)	0.4191 (3)	0.0504 (12)	
H39A	0.2915	0.4841	0.3890	0.060*	
C11	0.8581 (3)	0.8773 (4)	0.6034 (3)	0.0521 (13)	
H11A	0.8137	0.8375	0.6204	0.063*	
C31	0.1686 (3)	0.4212 (5)	0.5466 (3)	0.0596 (14)	
C22	0.9394 (3)	1.0752 (6)	0.3687 (3)	0.0764 (17)	
C26	0.1079 (3)	0.2602 (6)	0.4724 (5)	0.084 (2)	
H26A	0.0842	0.1825	0.4689	0.100*	
C42	0.1570 (3)	0.9229 (6)	0.3957 (4)	0.091 (2)	
H42A	0.1593	0.9249	0.3422	0.110*	
H42B	0.1628	1.0091	0.4142	0.110*	
C32	0.1309 (3)	0.3015 (5)	0.5413 (4)	0.0719 (18)	
C41	0.2134 (3)	0.8384 (4)	0.4258 (3)	0.0692 (15)	
H41A	0.2538	0.8341	0.3925	0.083*	
H41B	0.2297	0.8691	0.4734	0.083*	
C10	0.8134 (3)	1.1955 (5)	0.7035 (3)	0.0591 (14)	
C4	0.8831 (3)	1.2381 (5)	0.7176 (3)	0.0683 (16)	
H4A	0.8895	1.3137	0.7436	0.082*	
C38	0.3954 (2)	0.5194 (5)	0.4108 (3)	0.0522 (12)	
C3	0.9407 (3)	1.1739 (5)	0.6948 (3)	0.0665 (15)	
H3A	0.9860	1.2072	0.7030	0.080*	
C12	0.9237 (3)	0.6728 (5)	0.5805 (3)	0.0701 (16)	
H12A	0.9432	0.6894	0.5318	0.084*	
H12B	0.8778	0.6308	0.5754	0.084*	
C8	0.7341 (3)	1.0379 (5)	0.6519 (3)	0.0622 (14)	
H8A	0.7265	0.9630	0.6254	0.075*	
C30	0.1949 (3)	0.4570 (6)	0.6151 (4)	0.0794 (18)	
H30A	0.2211	0.5320	0.6193	0.095*	
C24	0.1520 (3)	0.4480 (5)	0.4139 (4)	0.0611 (14)	
C44	0.1058 (3)	0.7426 (5)	0.4580 (4)	0.0766 (17)	
H44A	0.1042	0.7514	0.5112	0.092*	
H44B	0.0726	0.6761	0.4433	0.092*	
C27	0.1212 (4)	0.2315 (6)	0.6069 (6)	0.098 (3)	
H27A	0.0967	0.1543	0.6042	0.118*	
C25	0.1188 (3)	0.3286 (5)	0.4107 (4)	0.0712 (16)	
H25A	0.1040	0.2962	0.3654	0.085*	
C19	0.8404 (3)	0.9221 (5)	0.3668 (3)	0.0645 (15)	
H19A	0.8360	0.9326	0.3160	0.077*	
C18	0.7966 (3)	0.8401 (5)	0.4044 (3)	0.0686 (15)	
H18A	0.7627	0.7929	0.3787	0.082*	
C37	0.4410 (3)	0.5868 (5)	0.4566 (3)	0.0708 (16)	
H37A	0.4901	0.5774	0.4517	0.085*	
C5	0.7527 (4)	1.2623 (5)	0.7266 (3)	0.0744 (16)	
H5A	0.7590	1.3384	0.7523	0.089*	
C15	0.9121 (3)	0.7469 (5)	0.7019 (3)	0.0729 (16)	
H15A	0.8640	0.7190	0.7131	0.088*	
H15B	0.9256	0.8138	0.7362	0.088*	
N2	0.9776 (4)	1.1433 (6)	0.3394 (3)	0.114 (2)	
C28	0.1449 (5)	0.2691 (8)	0.6720 (6)	0.113 (3)	
H28A	0.1365	0.2204	0.7141	0.136*	
C40	0.4246 (3)	0.4309 (6)	0.3597 (4)	0.0746 (17)	
C6	0.6855 (4)	1.2217 (7)	0.7135 (4)	0.0821 (18)	
H40A	0.6464	1.2691	0.7294	0.099*	
C7	0.6758 (3)	1.1065 (7)	0.6754 (4)	0.0794 (18)	
H7A	0.6300	1.0764	0.6661	0.095*	
C13	0.9750 (4)	0.5932 (6)	0.6269 (4)	0.103 (2)	
H13A	0.9643	0.5029	0.6222	0.123*	
H13B	1.0240	0.6077	0.6118	0.123*	
N4	0.4512 (3)	0.3616 (7)	0.3192 (4)	0.119 (2)	
C43	0.0872 (3)	0.8681 (6)	0.4207 (5)	0.103 (2)	
H43A	0.0558	0.8539	0.3789	0.123*	
H43B	0.0638	0.9254	0.4551	0.123*	
C29	0.1831 (5)	0.3845 (8)	0.6764 (4)	0.111 (3)	
H29A	0.2006	0.4117	0.7217	0.134*	
C14	0.9633 (4)	0.6377 (6)	0.7056 (4)	0.100 (2)	
H14A	0.9437	0.5692	0.7353	0.121*	
H14B	1.0081	0.6649	0.7274	0.121*	
C36	0.4140 (3)	0.6667 (5)	0.5085 (4)	0.0752 (17)	
H36A	0.4447	0.7127	0.5389	0.090*	

### Atomic displacement parameters (Å^2^)

**Table:**

	*U*^11^	*U*^22^	*U*^33^	*U*^12^	*U*^13^	*U*^23^
O1	0.060 (2)	0.066 (2)	0.090 (3)	−0.0146 (19)	0.007 (2)	−0.011 (2)
O2	0.081 (3)	0.067 (3)	0.079 (3)	−0.006 (2)	−0.009 (2)	−0.005 (2)
C20	0.055 (3)	0.046 (3)	0.052 (3)	0.000 (2)	−0.001 (3)	0.007 (2)
C34	0.051 (3)	0.041 (3)	0.052 (3)	−0.006 (2)	−0.002 (2)	0.001 (2)
C1	0.055 (3)	0.046 (3)	0.051 (3)	−0.010 (2)	0.003 (2)	0.000 (2)
N3	0.053 (2)	0.039 (2)	0.074 (3)	0.0024 (18)	0.012 (2)	−0.004 (2)
C17	0.050 (3)	0.063 (3)	0.071 (4)	−0.015 (3)	0.003 (3)	−0.009 (3)
C16	0.049 (3)	0.043 (3)	0.052 (3)	−0.004 (2)	0.002 (2)	−0.009 (2)
N1	0.065 (3)	0.047 (2)	0.054 (3)	−0.001 (2)	−0.002 (2)	0.002 (2)
C35	0.075 (4)	0.050 (3)	0.070 (4)	−0.005 (3)	−0.004 (3)	−0.011 (3)
C23	0.047 (3)	0.042 (3)	0.073 (3)	0.001 (2)	0.016 (3)	−0.001 (3)
C21	0.050 (3)	0.049 (3)	0.055 (3)	−0.007 (2)	0.000 (2)	−0.005 (2)
C33	0.064 (3)	0.035 (2)	0.055 (3)	−0.001 (2)	0.012 (3)	−0.002 (2)
C2	0.055 (3)	0.054 (3)	0.059 (3)	−0.009 (3)	0.010 (3)	−0.001 (2)
C9	0.059 (3)	0.048 (3)	0.041 (3)	−0.007 (2)	0.004 (2)	0.002 (2)
C39	0.052 (3)	0.044 (3)	0.055 (3)	−0.006 (2)	0.004 (2)	−0.005 (2)
C11	0.049 (3)	0.045 (3)	0.062 (3)	−0.009 (2)	−0.001 (2)	0.004 (2)
C31	0.061 (3)	0.041 (3)	0.077 (4)	0.003 (2)	0.023 (3)	−0.001 (3)
C22	0.088 (4)	0.084 (5)	0.057 (4)	−0.013 (4)	−0.007 (3)	0.006 (3)
C26	0.046 (3)	0.047 (3)	0.158 (7)	−0.004 (3)	0.008 (4)	−0.017 (5)
C42	0.090 (5)	0.056 (4)	0.129 (6)	0.017 (3)	0.001 (4)	0.007 (4)
C32	0.052 (3)	0.043 (3)	0.121 (6)	0.005 (3)	0.030 (4)	0.014 (3)
C41	0.076 (4)	0.042 (3)	0.090 (4)	−0.003 (3)	0.003 (3)	0.002 (3)
C10	0.068 (4)	0.052 (3)	0.058 (3)	−0.002 (3)	0.002 (3)	0.001 (3)
C4	0.084 (4)	0.056 (3)	0.065 (4)	−0.018 (3)	0.005 (3)	−0.010 (3)
C38	0.047 (3)	0.050 (3)	0.059 (3)	−0.005 (2)	0.007 (3)	0.004 (3)
C3	0.069 (4)	0.065 (3)	0.066 (4)	−0.022 (3)	0.004 (3)	−0.011 (3)
C12	0.090 (4)	0.054 (3)	0.067 (4)	0.009 (3)	−0.009 (3)	−0.003 (3)
C8	0.064 (4)	0.057 (3)	0.066 (4)	−0.004 (3)	0.004 (3)	0.001 (3)
C30	0.101 (5)	0.058 (4)	0.079 (5)	0.003 (3)	0.026 (4)	0.012 (4)
C24	0.049 (3)	0.049 (3)	0.085 (4)	−0.007 (2)	0.005 (3)	−0.015 (3)
C44	0.063 (3)	0.066 (4)	0.101 (5)	0.005 (3)	0.012 (3)	0.001 (3)
C27	0.086 (5)	0.049 (4)	0.160 (8)	0.008 (3)	0.048 (5)	0.029 (5)
C25	0.053 (3)	0.051 (3)	0.110 (5)	−0.005 (3)	0.000 (3)	−0.015 (4)
C19	0.071 (4)	0.064 (4)	0.058 (3)	0.001 (3)	−0.002 (3)	−0.005 (3)
C18	0.070 (4)	0.066 (4)	0.070 (4)	−0.012 (3)	−0.010 (3)	−0.014 (3)
C37	0.046 (3)	0.070 (4)	0.097 (5)	−0.007 (3)	−0.003 (3)	0.000 (3)
C5	0.094 (5)	0.065 (3)	0.064 (4)	0.006 (4)	0.007 (3)	−0.007 (3)
C15	0.096 (4)	0.062 (4)	0.061 (4)	−0.001 (3)	−0.011 (3)	0.002 (3)
N2	0.135 (5)	0.125 (5)	0.083 (4)	−0.047 (4)	0.015 (4)	0.016 (4)
C28	0.148 (8)	0.080 (6)	0.112 (7)	0.016 (5)	0.056 (6)	0.050 (5)
C40	0.045 (3)	0.090 (5)	0.089 (5)	0.003 (3)	0.007 (3)	−0.012 (4)
C6	0.084 (5)	0.081 (5)	0.081 (5)	0.026 (4)	0.003 (4)	−0.002 (4)
C7	0.060 (4)	0.092 (5)	0.086 (5)	0.007 (4)	0.004 (3)	0.007 (4)
C13	0.146 (7)	0.076 (4)	0.087 (5)	0.021 (4)	−0.015 (5)	0.003 (4)
N4	0.072 (4)	0.150 (6)	0.134 (6)	0.023 (4)	0.011 (4)	−0.041 (5)
C43	0.070 (4)	0.074 (4)	0.164 (7)	0.015 (3)	0.006 (5)	−0.002 (5)
C29	0.158 (7)	0.096 (6)	0.081 (5)	0.014 (5)	0.043 (5)	0.018 (4)
C14	0.136 (6)	0.067 (4)	0.098 (6)	0.013 (4)	−0.035 (5)	0.008 (4)
C36	0.071 (4)	0.061 (4)	0.094 (5)	−0.014 (3)	−0.014 (3)	−0.018 (3)

### Geometric parameters (Å, °)

**Table:**

O1—C2	1.357 (6)	C41—H41A	0.9700
O1—H1A	0.8200	C41—H41B	0.9700
O2—C24	1.339 (7)	C10—C5	1.400 (8)
O2—H2A	0.8200	C10—C4	1.403 (7)
C20—C19	1.388 (7)	C4—C3	1.336 (8)
C20—C21	1.391 (7)	C4—H4A	0.9300
C20—C22	1.441 (8)	C38—C37	1.384 (7)
C34—C35	1.382 (7)	C38—C40	1.419 (8)
C34—C39	1.389 (7)	C3—H3A	0.9300
C34—C33	1.523 (6)	C12—C13	1.526 (8)
C1—C2	1.381 (7)	C12—H12A	0.9700
C1—C9	1.421 (7)	C12—H12B	0.9700
C1—C11	1.537 (7)	C8—C7	1.376 (8)
N3—C33	1.469 (6)	C8—H8A	0.9300
N3—C44	1.477 (6)	C30—C29	1.363 (9)
N3—C41	1.481 (6)	C30—H30A	0.9300
C17—C18	1.381 (8)	C24—C25	1.399 (7)
C17—C16	1.395 (7)	C44—C43	1.519 (8)
C17—H17A	0.9300	C44—H44A	0.9700
C16—C21	1.381 (6)	C44—H44B	0.9700
C16—C11	1.520 (7)	C27—C28	1.322 (11)
N1—C11	1.471 (6)	C27—H27A	0.9300
N1—C12	1.487 (6)	C25—H25A	0.9300
N1—C15	1.491 (7)	C19—C18	1.369 (8)
C35—C36	1.381 (7)	C19—H19A	0.9300
C35—H35A	0.9300	C18—H18A	0.9300
C23—C24	1.395 (8)	C37—C36	1.357 (8)
C23—C31	1.415 (7)	C37—H37A	0.9300
C23—C33	1.531 (6)	C5—C6	1.351 (9)
C21—H21A	0.9300	C5—H5A	0.9300
C33—H33A	0.9800	C15—C14	1.494 (8)
C2—C3	1.423 (7)	C15—H15A	0.9700
C9—C8	1.403 (7)	C15—H15B	0.9700
C9—C10	1.433 (7)	C28—C29	1.408 (12)
C39—C38	1.389 (6)	C28—H28A	0.9300
C39—H39A	0.9300	C40—N4	1.147 (7)
C11—H11A	0.9800	C6—C7	1.402 (9)
C31—C30	1.388 (8)	C6—H40A	0.9300
C31—C32	1.442 (8)	C7—H7A	0.9300
C22—N2	1.142 (7)	C13—C14	1.516 (9)
C26—C25	1.343 (9)	C13—H13A	0.9700
C26—C32	1.389 (9)	C13—H13B	0.9700
C26—H26A	0.9300	C43—H43A	0.9700
C42—C41	1.482 (8)	C43—H43B	0.9700
C42—C43	1.497 (9)	C29—H29A	0.9300
C42—H42A	0.9700	C14—H14A	0.9700
C42—H42B	0.9700	C14—H14B	0.9700
C32—C27	1.409 (10)	C36—H36A	0.9300
			
C2—O1—H1A	109.5	C39—C38—C40	120.6 (5)
C24—O2—H2A	109.5	C4—C3—C2	120.1 (5)
C19—C20—C21	121.3 (5)	C4—C3—H3A	119.9
C19—C20—C22	120.1 (5)	C2—C3—H3A	119.9
C21—C20—C22	118.6 (5)	N1—C12—C13	102.5 (4)
C35—C34—C39	118.2 (4)	N1—C12—H12A	111.3
C35—C34—C33	120.0 (4)	C13—C12—H12A	111.3
C39—C34—C33	121.8 (4)	N1—C12—H12B	111.3
C2—C1—C9	119.1 (4)	C13—C12—H12B	111.3
C2—C1—C11	120.0 (4)	H12A—C12—H12B	109.2
C9—C1—C11	120.9 (4)	C7—C8—C9	122.8 (5)
C33—N3—C44	110.8 (4)	C7—C8—H8A	118.6
C33—N3—C41	114.9 (4)	C9—C8—H8A	118.6
C44—N3—C41	103.7 (4)	C29—C30—C31	121.4 (7)
C18—C17—C16	121.3 (5)	C29—C30—H30A	119.3
C18—C17—H17A	119.3	C31—C30—H30A	119.3
C16—C17—H17A	119.3	O2—C24—C23	122.8 (4)
C21—C16—C17	118.0 (5)	O2—C24—C25	117.6 (6)
C21—C16—C11	121.9 (4)	C23—C24—C25	119.7 (6)
C17—C16—C11	120.2 (4)	N3—C44—C43	105.3 (5)
C11—N1—C12	115.7 (4)	N3—C44—H44A	110.7
C11—N1—C15	112.6 (4)	C43—C44—H44A	110.7
C12—N1—C15	103.7 (4)	N3—C44—H44B	110.7
C36—C35—C34	121.5 (5)	C43—C44—H44B	110.7
C36—C35—H35A	119.2	H44A—C44—H44B	108.8
C34—C35—H35A	119.2	C28—C27—C32	123.7 (7)
C24—C23—C31	120.5 (5)	C28—C27—H27A	118.1
C24—C23—C33	119.8 (5)	C32—C27—H27A	118.1
C31—C23—C33	119.7 (5)	C26—C25—C24	120.6 (6)
C16—C21—C20	120.3 (5)	C26—C25—H25A	119.7
C16—C21—H21A	119.9	C24—C25—H25A	119.7
C20—C21—H21A	119.9	C18—C19—C20	118.4 (5)
N3—C33—C34	113.3 (4)	C18—C19—H19A	120.8
N3—C33—C23	109.1 (4)	C20—C19—H19A	120.8
C34—C33—C23	111.5 (4)	C19—C18—C17	120.8 (5)
N3—C33—H33A	107.6	C19—C18—H18A	119.6
C34—C33—H33A	107.6	C17—C18—H18A	119.6
C23—C33—H33A	107.6	C36—C37—C38	120.1 (5)
O1—C2—C1	123.4 (4)	C36—C37—H37A	120.0
O1—C2—C3	116.0 (5)	C38—C37—H37A	120.0
C1—C2—C3	120.6 (5)	C6—C5—C10	123.2 (6)
C8—C9—C1	124.0 (4)	C6—C5—H5A	118.4
C8—C9—C10	116.3 (5)	C10—C5—H5A	118.4
C1—C9—C10	119.7 (4)	N1—C15—C14	104.2 (5)
C34—C39—C38	120.1 (4)	N1—C15—H15A	110.9
C34—C39—H39A	119.9	C14—C15—H15A	110.9
C38—C39—H39A	119.9	N1—C15—H15B	110.9
N1—C11—C16	112.2 (4)	C14—C15—H15B	110.9
N1—C11—C1	109.8 (4)	H15A—C15—H15B	108.9
C16—C11—C1	111.4 (4)	C27—C28—C29	118.5 (7)
N1—C11—H11A	107.7	C27—C28—H28A	120.8
C16—C11—H11A	107.7	C29—C28—H28A	120.8
C1—C11—H11A	107.7	N4—C40—C38	176.9 (6)
C30—C31—C23	124.2 (5)	C5—C6—C7	118.6 (6)
C30—C31—C32	117.9 (6)	C5—C6—H40A	120.7
C23—C31—C32	117.8 (6)	C7—C6—H40A	120.7
N2—C22—C20	179.4 (7)	C8—C7—C6	119.9 (6)
C25—C26—C32	122.4 (5)	C8—C7—H7A	120.0
C25—C26—H26A	118.8	C6—C7—H7A	120.0
C32—C26—H26A	118.8	C14—C13—C12	105.1 (5)
C41—C42—C43	106.4 (5)	C14—C13—H13A	110.7
C41—C42—H42A	110.4	C12—C13—H13A	110.7
C43—C42—H42A	110.4	C14—C13—H13B	110.7
C41—C42—H42B	110.4	C12—C13—H13B	110.7
C43—C42—H42B	110.4	H13A—C13—H13B	108.8
H42A—C42—H42B	108.6	C42—C43—C44	105.4 (5)
C26—C32—C27	123.8 (7)	C42—C43—H43A	110.7
C26—C32—C31	118.8 (6)	C44—C43—H43A	110.7
C27—C32—C31	117.4 (7)	C42—C43—H43B	110.7
N3—C41—C42	104.8 (4)	C44—C43—H43B	110.7
N3—C41—H41A	110.8	H43A—C43—H43B	108.8
C42—C41—H41A	110.8	C30—C29—C28	121.1 (8)
N3—C41—H41B	110.8	C30—C29—H29A	119.5
C42—C41—H41B	110.8	C28—C29—H29A	119.5
H41A—C41—H41B	108.9	C15—C14—C13	106.6 (5)
C5—C10—C4	122.9 (5)	C15—C14—H14A	110.4
C5—C10—C9	119.0 (5)	C13—C14—H14A	110.4
C4—C10—C9	118.1 (5)	C15—C14—H14B	110.4
C3—C4—C10	122.4 (5)	C13—C14—H14B	110.4
C3—C4—H4A	118.8	H14A—C14—H14B	108.6
C10—C4—H4A	118.8	C37—C36—C35	120.0 (5)
C37—C38—C39	120.1 (5)	C37—C36—H36A	120.0
C37—C38—C40	119.1 (5)	C35—C36—H36A	120.0

### Hydrogen-bond geometry (Å, °)

**Table:**

*D*—H···*A*	*D*—H	H···*A*	*D*···*A*	*D*—H···*A*
O1—H1A···N1	0.82	1.90	2.576 (5)	139
O2—H2A···N3	0.82	1.93	2.593 (5)	138
C39—H39A···O2	0.93	2.58	3.292 (6)	133
